# Robust signal peptides for protein secretion in *Yarrowia lipolytica*: identification and characterization of novel secretory tags

**DOI:** 10.1007/s00253-018-8966-9

**Published:** 2018-04-27

**Authors:** Ewelina Celińska, Monika Borkowska, Wojciech Białas, Paulina Korpys, Jean-Marc Nicaud

**Affiliations:** 1 Department of Biotechnology and Food Microbiology, Poznan University of Life Sciences, ul. Wojska Polskiego 48, 60-627 Poznań, Poland; 2 INRA-AgroParisTech, UMR1319, Team BIMLip: Integrative Metabolism of Microbial Lipids, Domaine de Vilvert, Micalis Institute, 78352 Jouy-en-Josas, France

**Keywords:** Protein expression, Protein secretion, Signal peptides, Leader sequences, *Yarrowia lipolytica*, Modular cloning

## Abstract

Upon expression of a given protein in an expression host, its secretion into the culture medium or cell-surface display is frequently advantageous in both research and industrial contexts. Hence, engineering strategies targeting folding, trafficking, and secretion of the proteins gain considerable interest. *Yarrowia lipolytica* has emerged as an efficient protein expression platform, repeatedly proved to be a competitive secretor of proteins. Although the key role of signal peptides (SPs) in secretory overexpression of proteins and their direct effect on the final protein titers are widely known, the number of reports on manipulation with SPs in *Y. lipolytica* is rather scattered. In this study, we assessed the potential of ten different SPs for secretion of two heterologous proteins in *Y. lipolytica*. Genomic and transcriptomic data mining allowed us to select five novel, previously undescribed SPs for recombinant protein secretion in *Y. lipolytica*. Their secretory potential was assessed in comparison with known, widely exploited SPs. We took advantage of Golden Gate approach, for construction of expression cassettes, and micro-volume enzymatic assays, for functional screening of large libraries of recombinant strains. Based on the adopted strategy, we identified novel secretory tags, characterized their secretory capacity, indicated the most potent SPs, and suggested a consensus sequence of a potentially robust synthetic SP to expand the molecular toolbox for engineering *Y. lipolytica*.

**Electronic supplementary material:**

The online version of this article (10.1007/s00253-018-8966-9) contains supplementary material, which is available to authorized users.

## Introduction

Upon heterologous overexpression of a given protein in an expression host, its secretion into the culture medium is advantageous in both research and industrial contexts, since it greatly simplifies assaying its activity as well as its production/ purification. To target the nascent polypeptide to the secretion pathway, a signal peptide (leader sequence, signal sequence; hereafter abbreviated as SP) should be transcriptionally fused upstream of the mature sequence of a protein of interest (Madzak and Beckerich 2013). Such an N-terminus of a secretory protein may contain pre- or pre-pro-leader sequences. The pre-sequence is recognized by the signal recognition particle (SRP) receptors located on the surface of endoplasmic reticulum (ER) and thus is responsible for directing the polypeptide to translocation into the ER lumen, while the pro-leader, frequently bearing N-glycosylation sites, is involved in folding and maturation of the protein before it is packed into vesicles for exocytosis. The pro-sequence is also known to increase solubility of the protein inside the ER (Kjeldsen et al. 1998, 1999) and to traverse the cargo polypeptide in an inactive form during intracellular and inter-organelle transportation (Rakestraw et al. 2009). The pre-leader initiates ER translocation, and it is finally removed by the action of a signal peptidase in ER, while the pro-leader is processed in the Golgi compartment by the action of the KEX2, STE13, and KEX1 proteases or their homologs (like XPR6 in *Y. lipolytica*). It is also known that the pro-leader is not obligatory needed for heterologous protein secretion, while pre-leader is indispensable for secretion of majority of proteins. Accurate selection or design of a SP is considered the key factor affecting the secretory protein production rates (Ng et al. 1996; Rakestraw et al. 2009; Viña-Gonzalez et al. 2015; Yarimizu et al. 2015; Obst et al. 2017). It was demonstrated that pre-sequence alone is sufficient to specify the translocation pathway that is used by a protein (Fabre et al. 1991; Ng et al. 1996; Matoba and Ogrydziak 1998) ultimately determining its secretion rate.

*Y. lipolytica*, a non-conventional yeast species known for its unique metabolic properties, has emerged as an efficient platform for protein expression and secretion (Barth and Gaillardin 1996; Steinborn et al. 2005). So far, more than 130 heterologous proteins have been expressed in this host system, using a wide range of genetic engineering tools (Juretzek et al. 2001; Fickers et al. 2003; Nicaud 2012; Madzak and Beckerich 2013; Madzak 2015). By several criteria, *Y. lipolytica* was proven to be a far better secretor of proteins than the conventional yeast host—*Saccharomyces cerevisiae*—as it relies mainly on the co-translational translocation of the polypeptide to the ER lumen, being key strength of *Y. lipolytica* expression system, or exhibits higher biases in codon usage values for secretory pathway genes suggesting that these components could be expressed at higher levels in *Y. lipolytica* (Madzak et al. 2004; Ogrydziak and Nicaud 2012). The mechanisms driving translation, maturation, and secretion of the protein, as well as the response to increased secretory pathway cargo load in *Y. lipolytica*, were examined in great details in comprehensive studies on alkaline extracellular protease (AEP; *XPR2*), which became a model protein for this research area (Matoba et al. 1988; Fabre et al. 1991; He et al. 1992; Yaver et al. 1992; Le Dall et al. 1994; Ogrydziak and Nicaud 2012). Up to date, heterologous proteins expressed in *Y. lipolytica* cells have been mainly directed to the secretory pathway via *XPR2*-derived and *LIP2*-derived SPs, or their corresponding hybrids, while smaller proportion was expressed with their native SPs (Madzak and Beckerich 2013). XPR2-prepro region is by far the most widely studied SP, exploited for secretory overexpression of heterologous proteins in *Y. lipolytica*, targeting the polypeptide translocation through ER membrane co-translationally (Madzak 2015). A sole XPR2 pre-leader (so the SP per se) was proved to efficiently target the polypeptide to the secretory pathway, without the need for pro-leader (Nicaud et al. 1989; Fabre et al. 1991; Boisramé and Gaillardin 2009). Native LIP2 prepro SP (Pignede et al. 2000b), its hybrid with XPR2 pre-sequence (Nicaud et al. 2002), or synthetic SPs issued from the LIP2 pre region (Gasmi et al. 2011; Gasmi et al. 2012; Ledesma-Amaro et al. 2015) were all proved to operate with high efficiency in the secretory pathway of *Y. lipolytica*. Altogether, those studies demonstrated that a given heterologous protein could be correctly or incorrectly processed or expressed at several fold higher titers depending on the SP used. Consequently, in spite of great innate potential of *Y. lipolytica* cells towards secretory overexpression of proteins, modulation of the protein secretion rate can still be further enhanced when an appropriate SP is being adopted.

Although the key role of the SPs in secretory overexpression of heterologous proteins and its direct effect on the final protein titers are widely known, the number of reports on manipulation with SPs upon heterologous protein expression in *Y. lipolytica* is rather scattered. In this paper, we describe the first comprehensive study assessing capacity of ten different SPs (pre-sequences) for driving expression and secretion of two heterologous proteins in *Y. lipolytica* cells. The SPs under study cover those well-known, like preLip2 (Pignede et al. 2000a; Pignede et al. 2000b) or preXPR2 (Madzak et al. 1999), some previously described, like hybrid preLIP2 (Gasmi et al. 2011; Gasmi et al. 2012; Ledesma-Amaro et al. 2015) or insect-derived preSoAMY (Celińska et al. 2015), or novel, previously undescribed SPs in the context of recombinant protein secretion in *Y. lipolytica*. The novel SPs were identified through genomic DNA data mining, and their secretory capacity was assessed experimentally in comparison with known secretory tags. We took advantage of Golden Gate approach, for construction of expression cassettes, and micro-volume enzymatic assays, for functional screening of large libraries of recombinant strains. Based on the adopted strategy, we identified novel secretory tags, characterized their secretory capacity, indicated the most potent SPs, and suggested a consensus sequence of a potentially robust synthetic SP to expand the molecular toolbox for engineering *Y. lipolytica*.

## Materials and methods

### In silico analyses

Genomic DNA sequence of *Y. lipolytica* CLIB122 used in this study can be acquired from GRYC database (http://gryc.inra.fr/). Amino acid sequences of AEP and LIP2 N-terminal polypeptides are available in GRYC database or Nucleotide database at NCBI (https://www.ncbi.nlm.nih.gov/). Function of proteins encoded by the sequences serving as the SPs donors was determined using GRYC database or Nucleotide database at NCBI. *D* score values, discriminating signal peptides form non-signal peptides based on probability of the presence of a signal peptidase cleavage site, as well as the primary amino acid structure of the SPs were predicted using SignalP 4.1 (Petersen et al. 2011) (http://www.cbs.dtu.dk/services/SignalP/) and PrediSi (Hiller et al. 2004) (http://www.predisi.de/) tools. Hydrophobicity of the SP sequences was assessed using the grand average of hydropathy (GRAVY) calculator (http://www.gravy-calculator.de/) for the stretch of 12 amino acid residues after the last positively charged residue (HB12 value) or for the whole SP sequence prior to the signal peptidase cleavage site. For the SP9, where no positively charged amino acid residue was present at the N-terminus, two HB12 values were calculated: (i) for the 12 amino acids directly after N-terminal methionine and (ii) for the 12 amino acids forming an alpha-helix, as determined by secondary structure analysis. Secondary structure of the SPs was predicted using SOPMA tool (secondary structure prediction method; https://npsa-prabi.ibcp.fr/cgi-bin/npsaautomat.pl?page=/NPSA/npsasopma.html; (Combet et al. 2000)). Alignment of the most robust SPs was done using MEGA 7.0.14 package and ClustalW algorithm (Kumar et al. 2016). The consensus sequence and its logo were determined using Web Logo tool at http://weblogo.berkeley.edu/logo.cgi.

### Strains and routine culturing conditions

All strains and plasmids used in this study are listed in Online Resource ESM_1 and Online Resource ESM_2. All the cultivations required for molecular biology protocols complied with the standards described in Barth and Gaillardin (1996) and Sambrook and Russell (2001). Briefly, *Escherichia coli* strains were routinely maintained in LB medium (liquid or solidified with agar) supplemented with appropriate antibiotic when necessary, at 37 °C, 250 rpm. *Y. lipolytica* strains were routinely grown in YNB or YPD media (liquid or solidified with agar), at 28 °C, 250 rpm.

### Molecular biology protocols

If not stated otherwise, all the molecular biology protocols followed the methodologies described in Sambrook and Russell (2001). All oligonucleotides and longer synthetic DNA fragments used in this study are listed in Online Resource ESM_3. *E. coli* and *Y. lipolytica* transformation protocols were conducted according to the heat-shock or LiAc methodologies described in Sambrook and Russell (2001) and Barth and Gaillardin (1996), respectively. Genomic DNA isolation from *Y. lipolytica* cells, plasmid isolation from *E. coli*, DNA fragment extraction from agarose gel, or purification of DNA fragments were all conducted using appropriate kits from A&A Biotechnology (Poland)—Genomic Mini AX Yeast, Plasmid Mini, Gel-Out, Clean-Up. Restriction digestion of DNA fragments was done using either *NotI* enzyme (Thermo Scientific) or *BsaI* (New England Biolabs). Routine colony PCR with *E. coli* biomass was conducted using Taq DNA polymerase RUN (A&A Biotechnology; Poland), while colony PCR with *Y. lipolytica* biomass was conducted using Phire Hot Start II DNA Polymerase (Thermo Scientific). Phire Hot Start II DNA Polymerase was also used for amplification of all Golden Gate Fragments (GGFs; amplicons constituting individual biobricks in Golden Gate Assemblies) used in this study. All the reactions were conducted according to the protocols provided by the manufacturers.

### Modular cloning—Golden Gate Assembly and positive clone selection

The modular cloning protocol followed a previously set standard (Celinska et al. 2017). Briefly, a set of thirteen 4-nt overhangs, matching the corresponding destination vector pSB1A3-RFP, available from iGEM collection (http://parts.igem.org/Collections), was developed. In this study, the scaffold could be narrowed to a single transcription unit-bearing variant, altogether comprising (overhangs are marked in bold): -**A**-Insertion UP (zeta_*NotI*)-**B**-selection Marker (URA3)-**C**-promoter P1 (pTEF)-**D**-signal peptide SP (10 biobricks)- **X** (novel 4 nt overhang) + gene of interest (*SoAMY* or *TlGAMY*)-**K**-terminator T3-**L**-Insertion DOWN (zeta_*NotI*)-**M**-. A novel 4-nt overhang “X” was developed here to fit the previously designed scaffold and to leave the SPs’ amino acid sequence possibly unchanged, which was the key prerequisite upon this overhang design. Based on analysis of the nucleotide sequences coding for SPs, an overhang TGCC was established as the most optimal sequence. The SPs were synthesized as complete GGFs flanked with “D” and “X” overhangs and the *BsaI* recognition sites, and cloned in the donor vector. All the Golden Gate Vectors (GGVs; GGF-bearing donor vectors) were constructed using pCR Blunt II TOPO vectors (Thermo Scientific), according to the instruction provided by the manufacturer. Golden Gate reaction mixtures contained precalculated equimolar amounts of each GGF and the destination vector (50 pmol of ends), 2 μL of T4 DNA ligase buffer (NEB), 5 U of *BsaI*, 200 U of T4, and ddH_2_O up to 20 μL. The following thermal profile was applied: [37 °C for 5 min, 16 °C for 2 min] × 30, 80 °C for 5 min, 15 °C ∞. The limited number of the biobricks covered by the complete assemblies designed in this study allowed to decrease the number of digestion-ligation cycles from 60, as previously applied, to 30. Subsequently, the reaction mixtures were used for *E. coli* JM109 transformation. White colonies were screened for identification of complete Golden Gate Assembly (GGA) through colony PCR, followed by plasmid isolation, restriction digestion, and multiplex PCR. Complete GGAs were subsequently linearized with *NotI* endonuclease and used for transformation of *Y. lipolytica* JMY2101 strain. Clones appearing after 48 h incubation at 30 °C on YNB-selection plates were replica-plated on fresh YNB, YPD, and YPS agar plates (g/L: yeast extract, 10; bacto peptone, 20; glucose, 20; starch, 10; agar, 15). All the clones were screened for the GGA presence through colony PCR. Moreover, the clones were screened via starch-iodine drop test, as described previously (Celińska et al. 2016b). Briefly, after 48-h culturing on YPS plates, the biomass was scraped and 5% iodine solution (I2 in KI) was poured onto the plate to visualize the translucent zones. All the strains bearing the GGA and generating translucent zones in the starch-iodine drop test were deposited as glycerol stocks at − 80 °C. Altogether, more than 400 *Y. lipolytica* strains bearing 20 different variants of GGA were obtained. Five clones out of each variant were subjected to cultivation and enzymatic activity tests.

### Enzymatic activity tests—microSIT and microSNT protocols

Five representative strains bearing a respective GGA variant were cultured in shake flask batch cultivations in YPG medium (g/L: yeast extract, 10; bacto peptone, 20; glycerol, 20; buffered at pH 5.7 with 100 mM phosphate buffer; total flask volume 100 mL, culture medium volume 15 mL), at 28 °C, 250 rpm in a rotary shaker incubator (Biosan). The following activity tests were conducted according to a previously designed methodology: for SoAMY alpha-amylase-microSIT, and for TlGAMY glucoamylase-microSNT, described in detail in Borkowska et al. (in press). Briefly, a Verity 96-well Thermal Cycler (Applied Biosystems) was used for incubation and heating of the samples. The enzymatic microassays were performed in 96-well semi-skirted PCR plates (4-titude, UK) tightly covered with microplate sealing mats (Axymat, Axygen). Solution of rice starch (Sigma-Aldrich; 2 g/L) in acetate buffer (100 mM, pH 5.0) was used as the substrate and combined with an equal volume of the sample containing the enzyme (culture medium supernatant). Incubation of the substrate and the enzyme-containing sample (1:1) was continued for 120 min at 40 °C. In the microSNT assay, the reactions were stopped by the addition of one volume of the Nelson’s copper reagent A + B and heated up to 99.9 °C for 5 min. Samples were cooled down to the room temperature and mixed with half volume of the arsenate-molybdate reagent. In the microSIT assay, the reaction was stopped by adding one fourth volume of 1 M HCl, and the remaining starch was stained with one volume of iodine solution. Completed reaction mixtures were subsequently transferred into a transparent flat-bottomed 96-well assay microplate (Corning) and analyzed using a Tecan Infinite M200 automatic plate reader, measuring the absorbance of the samples (wavelength, 660 nm–SNT; 580 nm–SIT). For normalization, the sugar background controls in the microSNT assay or undigested starch control in the microSIT assay (the substrate plus the sample without incubation—controls were stopped prior to addition of the sample) were run simultaneously and allowed for in the enzyme activity calculations. Moreover, upon each of the three independent runs of the recombinant strains batch cultivations, cultivation of a positive control strain was run in parallel. The positive controls were *Po1g* strains expressing either *SoAMY* or *TlGAMY* gene under the control of the p*hp4d* promoter and equipped with the spXPR2 from pYLSC vector (YLEX Expression kit; Yeastern Biotech Co., Ltd., Taiwan). The test-specific reagents were prepared as follows: Nelson’s copper reagent A (g/L): sodium carbonate anhydrous, 25; sodium-potassium tartrate tetrahydrate, 25; and sodium sulfate, 200; Nelson’s copper reagent B (g/L): copper sulfate pentahydrate, 150 and concentrated sulfuric acid, 20 drops; prior the enzymatic reaction, the reagents A and B were mixed in proportion 25:1; arsenate-molybdate reagent (g/L): sodium molybdate dihydrate, 50; concentrated sulfuric acid, 42 mL; sodium arsenate dibasic heptahydrate, 6; solution incubated at 37 °C, over 24–48 h prior to use; iodine: 5 mM I2 in 5 mM KI and 1 M HCl for stopping the reaction in microSIT assay. In order to compare operability and secretory capacity of the SPs under study for the two amylolytic enzymes, assayed through the different protocols, the results were presented as relative values with respect to the applied positive controls. Such an approach allowed to compare the results irrespective of the differences in the assaying conditions. All the cultures were done in three independent runs for the five representative strains. Each sample was processed in technical duplicate.

### Statistical analysis

The results of the enzymatic activity tests were expressed as means ± standard deviations of the means (means ± SD) from the replicates, as mentioned above. Parametric analyses (one-way ANOVA) were used for the data processing. Distributional assumptions for applying ANOVA analyses were assessed by the Shapiro-Wilk test, while homogeneity of variances between the subjects was assessed using Levene’s tests. When the normality assumption was questionable and the variance dishomogeneity was present, the normalizing transformations were performed. The following series of transformations was used: logarithmic, inverse, and square root. Consequently, as indicated by the performed analyses, the experimental data should be described by a square root transformation (SQRT). Scheffe’s test was used as a post hoc test, provided that the significance was detected between the variants at the level of *p* < 0.05. Statistical analyses were performed with the STATISTICA data analysis software system (StatSoft, Inc., Tulsa, OK, USA). The results were considered to be statistically different at a *p* value of 0.05 or less.

## Results

### Identification of novel SPs, design of expression cassettes, and computational analyses of the SP characteristics

**Fig. 1:**
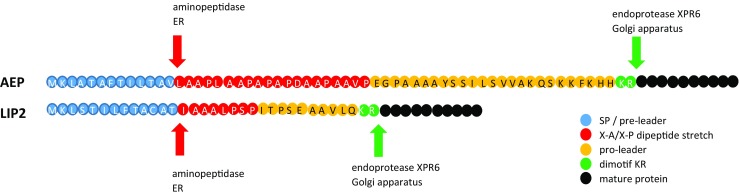
Signal peptides of AEP and LIP2—the major proteins of *Y. lipolytica* secretome. Color code of the scheme is explained in legend: blue—pre-leader sequence, red—XA/XP dipeptide stretch, yellow—pro-leader, green—dimotif KR, black—mature protein. Arrows indicate sites recognized by specific aminopeptidase (red) operating in the endoplasmic reticulum (ER) or by endoprotease XPR6 (KEX2 homolog) (green), operating in the Golgi apparatus

Identification of the novel SPs was conducted through *Y. lipolytica* CLIB122 genome-sequence scanning for occurrence of a MK_x{7–15}_(x,A/P)_{2–10}_x{10–120}_KR motif, where the pre-sequence is composed of a stretch of 7 to 15 amino acid residues of any type “x{7–15}” preceded by an “MK” element and followed by 2 to 10 repetitions of a dipeptide motif -X-A- terminated with P residue “(x,A/P)_{2–10}_”, while the pro-sequence consists of 10 to 120 amino acids terminated with a KR element. The motif was inferred from analysis of amino acid N-terminal sequences of two major secretory proteins in *Y. lipolytica*—AEP and LIP2, expressed as prepro proteins. The structure of the prepro regions and maturation process of the two proteins is schematically presented in Fig. 1. Altogether 54 proteins (Online Resource ESM_4) initialized by the indicated motif were identified (Neuvéglise 2008, unpublished). From among these, we eliminated 16 proteins, where the presence of an SP was assessed as not statistically significant by SignalP tool. The remaining 38 N-terminal polypeptides (covering the pre-sequence solely) were aligned and the consensus sequence was presented in a form of logo (Table 1.A), corresponding to a SP typical for secretory proteins in *Y. lipolytica*. Based on the adopted genome-mining methodology as well as accessory transcriptomic analyses (not shown), we proposed five novel SPs, native for secretory proteins highly expressed in *Y. lipolytica*. The proteins are annotated with the following numbers in the genome: YALI0B03564g (SP1), YALI0D20680g (SP2), YALI0E22374g (SP3), YALI0D06039g (SP4), and YALI0D06149g (SP5) (the functions indicated in Table 2). For comparative purpose, well-known SPs, widely applied in secretory expression of proteins in *Y. lipolytica*, were included in this study, native spLip2 (SP6) (Pignede et al. 2000ab), and its engineered variant, spLip2pre-3xLA (SP7) (Ledesma-Amaro et al. 2015), as well as preXPR2 (SP10) (Matoba et al. 1997). Additionally, native SPs of the two proteins under study SoAMY (SP8) (*Sitophilus oryzae* alpha-amylase) and TlGAMY (SP9) (*Thermomyces lanuginosus* glucoamylase) (both sequences were codon optimized) were also investigated (Table 2).

**Table 1:**
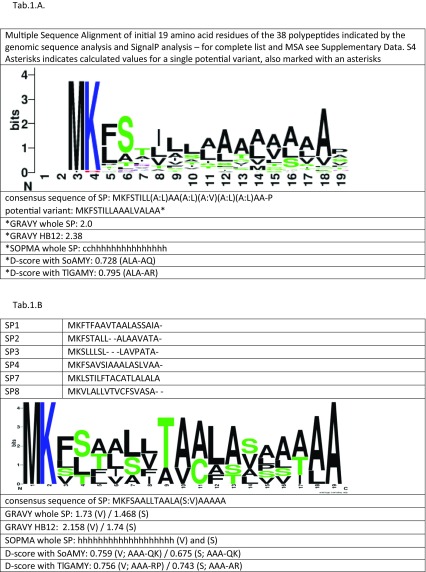
Signal peptide consensus sequence logos, secondary structure prediction, hydrophobicity assessment, and *D* score values for (**a**) the 38 proteins identified through *Y. lipolytica* CLIB122 genomic sequence scan for ORFs bearing MK x{7–15} (x,A/P){2–10} x{10–120} KR motif and (**b**) SPs having the highest secretory capacity in *Y. lipolytica* cells with SoAMY and TlGAMY polypeptides

Amino acid sequences were aligned using MEGA 7.0.14 package and ClustalW algorithm; dashes indicate gaps; ambiguous sites are indicated as (X:Y) X and Y—alternative amino acid residues occurring with the same frequency. The logos were created using Web Logo tool. Color code: black—hydrophobic amino acid residue, blue—positively charged amino acid residue, green—polar uncharged amino acid residue. Size of a letter represents frequency of a corresponding amino acid residue occurrence in a respective position. Hydrophobicity evaluation was done using GRAVY calculator tool and secondary structure prediction—using SOPMA tool. (A) Due to relatively high sequence degeneration SOPMA and GRAVY analyses were done for one possible variant; (B) Both analyses were done for the two possible variants of the consensus sequence

**Table 2:**
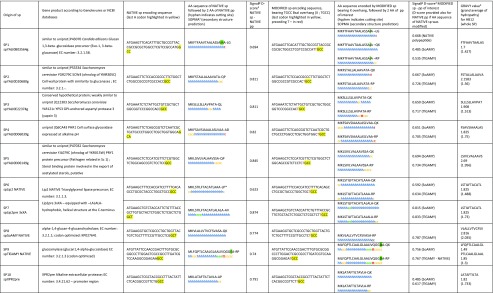
Signal peptides under study accompanied by the results of computational analyses

^a^SOPMA: prediction of secondary structure; : alpha-helix,: beta turn, : random coil, : extended strand

^b^*D* score is used to discriminate signal peptides from non-signal peptides based on probability of the presence of a signal peptidase cleavage site; SignalP

^c^GRAVY value: grand average hydropathy calculated for 12 residues after the last positively charged residue of the n-region (HB12) or complete SP

^d^SignalP calculates signal peptide of 27 AA (MKLSTILFTACATLAAALPSPITPSEA-A V); PrediSi tool predicts the pre region of 17 AA (as reported in primary scientific papers (Pigniede et al., 2000), and this pre-sequence was analyzed in this study

**Fig. 2:**
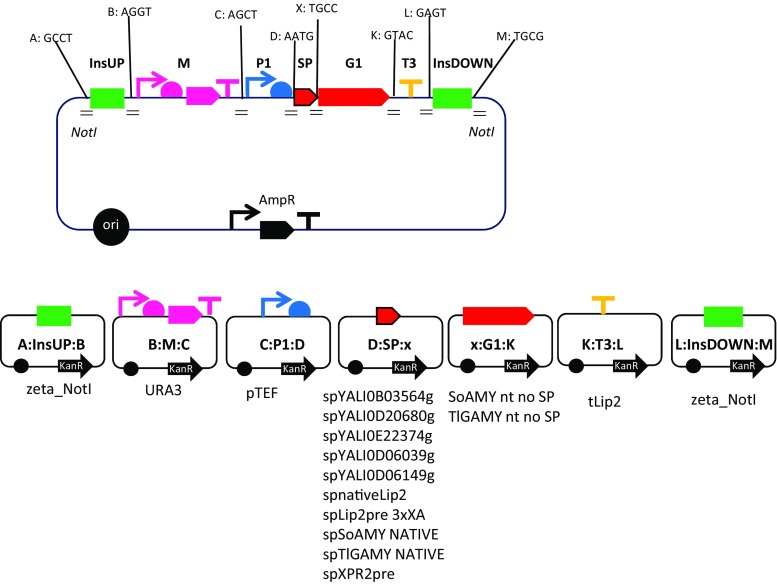
Golden Gate Assembly Scaffold. The scheme illustrates a scaffold of GGVAs designed and constructed in this study (Golden Gate Vectors bearing complete Assemblies; pSB1A3 backbone) alongside with Golden Gate donor Vectors (GGVs; TOPO backbone) bearing individual elements (Golden Gate Fragments (GGFs)) to be ultimately assembled into different GGVA variants, depending on the GGFs type. The complete GGVA bears seven elements: InsUP/InsDOWN—zeta elements serving as the target regions for non-homologous recombination events flanked with *NotI* recognition sites; M—selection marker *URA3*; P1—promoter *pTEF1*; SP—signal peptide, one of the ten SPs under study; G1—gene of interest, one of the two *SoAMY* or *TlGAMY* devoid of their native SP; T3—terminator *tLip2*. Symbols corresponding to GGFs follow Synthetic Biology Open Language (SBOL) standard. Each GGF is flanked with a 4-nt overhang assigned a respective letter and a 4-nt sequence indicated in the scheme (A, B, C, D, X, K, L, and M). Altogether 20 complete GGVAs were obtained. Prior to transformation GGA was released from GGVA through digestion with *NotI* endonuclease

The ten selected SPs were subsequently transcriptionally fused upstream of the two polypeptides under study. We adopted redesigned Golden Gate scaffold (Celinska et al. 2017) in order to facilitate cloning of an additional biobrick encoding different variants of the SPs between the promoter and the gene of interest. To this end, we included a novel 4-nt overhang (assigned a letter x) to fit into the previously developed scaffold and followed the strategy presented in (Fig. 2). Importantly, this novel overhang was designed in a way to maintain the SP’s amino acid sequence possibly unchanged. Prior to actual cloning of the genes of interest equipped with the selected SPs, the assemblies (SP-polypeptide) were in silico analyzed with their native polypeptides and one of the targeted amylolytic proteins (SignalP 4.1, PrediSi) to determine *D* score values assessing probability of digestion by a signal peptidase. Furthermore, secondary structure prediction (SOPMA; (Combet et al. 2000)) and calculation of general average hydrophobicity for H12 and the whole SPs (GRAVY values) were done for all the SPs variants under study. All the results are provided in Table 2.

### Assaying secretory capacity of the SPs upon overexpression of the amylolytic proteins in *Y. lipolytica* cells

Altogether 20 Golden Gate assemblies, differing in the SP sequence and the following gene of interest, were obtained in a course of the adopted methodology (Fig. 2). Correct assemblies were selected from *E. coli* clones. The corresponding plasmids were digested with *NotI* endonuclease and the expression cassettes were transformed into *Y. lipolytica* JMY2101 strain. Altogether, more than 400 clones were screened for integration of the cassette through colony PCR and starch-iodine drop test. The clones bearing the heterologous genes *SoAMY* and *TlGAMY*, and generating translucent halos on starch-containing plates stained with iodine, were deposited as glycerol stocks for long-term storage.

**Fig. 3:**
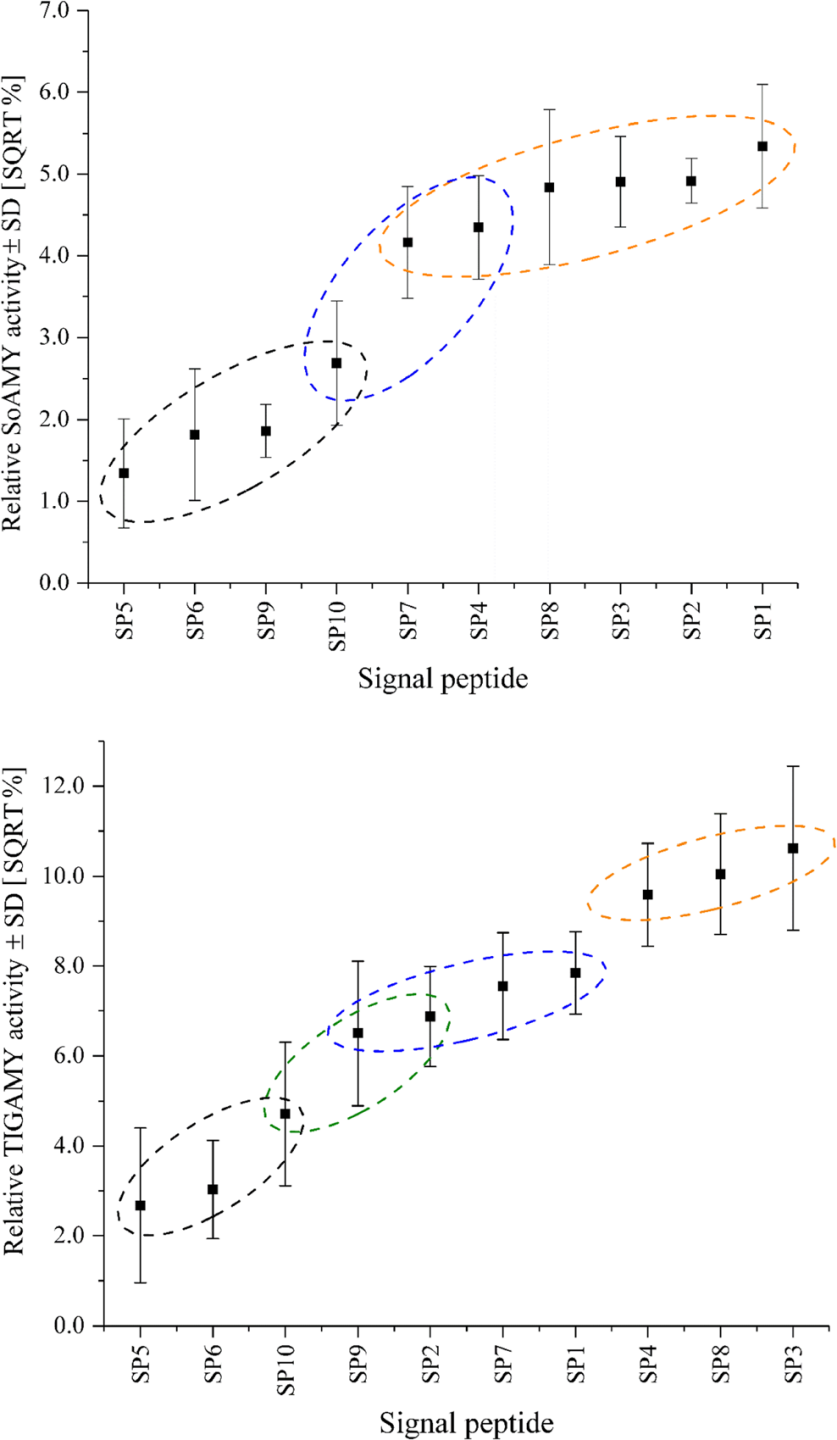
Relative amylolytic activity of SoAMY and TlGAMY in the batch culture medium supernatants of recombinant *Y. lipolytica* strains, transformed with GGAs bearing different SP-encoding sequences. The values constitute a mean from three independent runs of five subclone cultures, representing a corresponding GGA variant. The amylolytic activity was expressed as a SQRT% value in relation to the positive control strain (derivative of *Po1g* bearing pYLSC-*SoAMY*/*TlGAMY*; see Online Resource ESM_2). Grouping of the SPs under study depending on their secretory capacity towards SoAMY and TlGAMY proteins was conducted using Scheffe test. Error bars represent ±SD of three independent runs of five subclones representing a given GGA variant

Five representative subclones were subsequently subjected to cultivation tests followed by determination of the enzymatic activity of SoAMY and TlGAMY through microassays (Borkowska et al. in press). The results are shown in Fig. 3. With respect to secretion of SoAMY protein to the culture medium, the SP derived from YALI0D06149g (SP5), the native SP from spLip2 (SP6), and the SP from TlGAMY (SP9) turned out to be inferior when compared with the remaining SPs. In contrast, the highest SoAMY activity was detected in the culturing media of strains bearing GG assemblies with SP1, SP2, SP3, and SP8, followed by SP4 and SP7. From these data solely, it could be inferred that four out of five novel SPs compare favorably in terms of SoAMY secretion to the SPs spLip2 native (SP6) or spXPR2 (SP10), widely applied in the secretory protein overexpression in *Y. lipolytica*. The insect SP from SoAMY operated equally well as the novel SPs native for *Y. lipolytica* cells. Yet, the compatibility of the SP8 has been already previously discussed in detail based on in silico analyses and evidenced experimentally (Celińska et al. 2015; Celińska et al. 2016a; Celińska et al. 2016b). Strikingly, the SPs under study worked with corresponding efficiency for both proteins, as demonstrated by Pearson correlation coefficient (*r* = 0.835), indicating the dominant (but not the sole) role of the SP itself and not the following protein on the observed variation in this experiment. Regarding the extracellular TlGAMY activity results, the highest values were observed for SP3, SP8, and SP4 followed by SP1, SP7, and SP2. Expectedly, TlGAMY could be relatively well expressed and secreted with its native SP, which in contrast performed poorly with SoAMY protein. Again, SP5 and SP6 turned out to be the least suited for secretory overexpression of TlGAMY protein from among the tested SPs. The SPs could be clustered depending on their secretory efficiency with SoAMY and TlGAMY, as depicted in (Fig. 3). According to correlation analysis, we have not observed any significant positive correlation between experimentally obtained data (extracellular activity of a given enzyme) and in silico computed *D* score values (Pearson *r* = 0.22472). Correspondingly, we could not see any straightforward correlation between the hydrophobicity of the HB12 (Pearson *r* = 0.0258) or whole SP (Pearson *r* = 0.0131) and the final extracellular amylolytic activity value (neither SoAMY nor TlGAMY).

## Discussion

Upon heterologous overexpression of a protein, its secretion into the extracellular environment rather than accumulation inside the cell is a superior strategy. Considerable interest in a native extracellular proteome of a cell, resulted in development of a number of strategies facilitating determination of the pool of proteins creating the cell secretome, providing a great deal of tools and knowledge to be adopted in heterologous protein overexpression. Among the experimental methodologies designed for the secretome studies, one can name secretion traps or signal sequence traps, mass spectrometry, or Serial Analysis of Gene Expression (SAGE), as thoroughly discussed in Mukherjee and Mani (2013). A selection of computational methods enabling prediction of putative secretory proteins based on SP-encoding sequence occurrence in a given ORF was also developed. Advanced computational tools allow to predict the secretory proteins traversing via the classical pathway, like SignalP (Petersen et al. 2011), or those lacking a conventional SP, processed through a non-classical pathway, like SecretomeP (Bendtsen et al. 2004). In this study, we have adopted systematic analysis of *Y. lipolytica* strain CLIB122 complete genome (Dujon et al. 2004) for ORFs containing putative SP, inferred from a consensus structure of the two major secretory proteins of *Y. lipolytica* cells—AEP and LIP2. The SP-encoding regions of the selected proteins (Table 2) were analyzed in all the selected ORFs using SignalP (Petersen et al. 2011) and PrediSi (Hiller et al. 2004) tools to predict the extent of the leader domain and to assess probability of cleavage by signal peptidase (Table 2).

According to the literature data, e.g., Yang et al. (2006), Yarimizu et al. (2015)), a typical structure of a “Sec-type” yeast signal peptide covers (i) N-domain bearing at least one positively charged AA residue (R or K) and (ii) H-domain (hydrophobic core) built by a tract hydrophobic AA residues (e.g., A, L, V, F, C, Y, W, I, M) forming an alpha-helix, which is essential for translocation of the polypeptide through cellular membrane, terminated with (iii) C-domain: “helix-breaking” or polar (P, E, or G) residue, facilitating digestion through a specific signal peptidase, ended with a consensus sequence A-X-A (X-any residue), recognized by the specific signal peptidase. Although a general secondary structure of an operable SP was established, a strict consensus sequence has not been determined, as individual elements building an SP are highly variable in length and have no obvious sequence homology. Several comprehensive studies on the impact of individual site mutation in the N-terminal sequence on the secretory potential of the SP operating in a particular host system have been reported to date (Rakestraw et al. 2009; Viña-Gonzalez et al. 2015; Yarimizu et al. 2015). In the SPs under study, the first component (positively charged N-terminus) of a typical SP was identified in all the selected signal peptides but one (sp TlGAMY NATIVE), while the second determinant—hydrophobic alpha-helix—was found to be a common structural element, as the hydrophobic residues were abundant in the central region of all the peptides. The crucial importance of the N-terminal positively charged amino acid (K, but also operability of R, N, W, and F) and the following stretch of hydrophobic core in a SP was elegantly demonstrated in a comprehensive study by Yarimizu et al. on heterologous proteins expressed in *Kluyveromyces marxanius* cells (Yarimizu et al. 2015). In that paper, serial deletion of individual amino acids was conducted and the effect of a corresponding deletion was subsequently studied. It was concluded that the range of the crucial hydrophobic core was defined by the N-terminal basic and C-terminal non-hydrophobic amino acids (e.g., E or P). Clear marking of the boundaries was essential for the SP operability; however, the length of the hydrophobic core was different for each individual polypeptide, and optimal length of the hydrophobic helix could be determined for each of proteins analyzed in that study (having bacterial, fungal, and human origin). In this study, prediction of the cutting site in the SPs under study was first conducted for a given SP followed by its own native polypeptide sequence. The output SignalP *D* scores (column NATIVE sp–NATIVE pp in Table 2) can roughly reflect the secretory potential of the SP (*D* score is used to discriminate SPs from non-SP sequences by the secretion machinery with a given confidence). Based on these computational analyses, the engineered spLip2pre-3xLA SP (Ledesma-Amaro et al. 2015), equipped with a hydrophobic dipeptide stretch (-LALALA-), was identified as SP processed by signal peptidase with the highest confidence (*D* score 0.874), while its un-engineered counterpart (spLip2 native) was identified as SP with the weakest confidence out of the analyzed SPs (*D* score of 0.623). For all the remaining SPs equipped with their native polypeptides, the calculated *D* score values were between these two border values (Table 2).

With the advent of modular cloning strategies, like Gibson assembly, Golden Gate, or Gateway, the genetic engineering toolboxes have greatly expanded, offering much higher versatility, comprehensiveness, and high throughput character of the research. Adaptation of a given sequence as a “biobrick” to a modular cloning strategy requires fitting the sequence into a pre-designed scaffold. Since all of the in silico predicted sites recognized by a signal peptidase were preceded by an alanine residue encoded by, i.e., GCC codon, this motif was included in the novel x overhang. Analysis of the penultimate codon sequence allowed to state that T nucleotide in the first position of the 4-nt overhang is the optimal choice, as then the least number of SP amino acid sequences had to be changed. Nevertheless, in two cases (YALI0B03564g and spTlGAMY NATIVE), the amino acid residue directly prior to the terminal alanine had to be modified due to introduction of the T nucleotide at the third position of the penultimate codon (M ➔ I and E ➔ D, respectively; see Table 2). It was presumed that a change is admissible provided that the character of the encoded amino acid is maintained (hydrophobic ➔ hydrophobic and negatively charged ➔ negatively charged, respectively). Consequently, all the SP-encoding sequences were terminated with a TGCC overhang accompanied by a *BsaI* cutting site in an appropriate orientation to comply with the previously adopted standard. Such SPs were subsequently in silico equipped with the amino acid sequences of the two amylolytic enzymes under study (SoAMY or TlGAMY) devoid of their native signal sequences. Such hybrid sequences were again subjected to in silico prediction of the cutting site by signal peptidase (column MODIFIED sp-pp of interest; Table 2). In the case of the two modified SPs (YALI0B03564g and spTlGAMY NATIVE), the computational analysis was additionally conducted again with their native polypeptides. In the case of YALI0B03564g, the necessary change (M ➔ I) slightly decreased the *D* score value, while in the case of spTlGAMY NATIVE, the change worked the opposite. As shown in Table 2, depending of the combination of the SP and the following protein, the *D* score values could be either higher or lower with the SoAMY or TlGAMY (MODIFIED ss–pp of interest) when compared to these values obtained with their native polypeptides (NATIVE ss–NATIVE pp), indicating the importance of the global structure of a given SP covering all, the positively charged N-terminus, the hydrophobic core, and the polar C-terminus, which differed depending of the secreted protein (QK for SoAMY, RP for TlGAMY). Yet, the engineered spLip2pre-3xLA was again indicated as a SP with the highest confidence (*D* scores of 0.815 and 0.833 for SoAMY and TlGAMY, respectively), followed by spSoAMY leader sequence.

It has been reported that the average hydrophobicity of a given SP is an important determinant of whether the protein is targeted to the SRP-dependent or SRP-independent secretory pathway and if it will be translocated co- or post-translationally (Ng et al. 1996). It has been evidenced that by calculating the hydrophobicity values for each amino acid, a putative, optimal signal sequence can be predicted by computational methods (Kyte and Doolittle 1982; Hiller et al. 2004; Petersen et al. 2011). In a study by Yarimizu et al., it was demonstrated that it is rather a structure than a strict sequence being the determinant for the SP operability and that an effective SP requires an adequate hydrophobic core with a defined length and characterized by an optimal hydrophobicity value (Yarimizu et al. 2015). Moreover, it was evidenced that substitutions of glycines into leucines within the hydrophobic core of the SP (increasing hydrophobicity) resulted in improved interaction between an SP and SRP. However, increased hydrophobicity over a hydrophobicity threshold value was harmful for the SP function (Yarimizu et al. 2015). It is also known that individual SPs exhibit different levels of secretory potential in a given species, suggesting a preference towards a particular SP structure amongst different organisms. It was calculated that in *S. cerevisiae*, the proteins traversing via SRP-independent pathway should have the average hydrophobicity of the 12 residues after the last positively charged residue (HB12 value) of around 2.0 or less, while for the proteins targeted primarily by an SRP-dependent pathway, the HB12 values should be significantly higher, around 3.0 or more (Ng et al. 1996). However, further studies within this field clearly demonstrated that the hydrophobicity cannot be the sole factor driving this phenomenon (Matoba and Ogrydziak 1998). For example, it was shown that secondary structure was an important determinant influencing formation of a productive complex between isolated SPs and SRP. Beta-structure and random or unordered structures were favored in aqueous solution while alpha-helix in the nonpolar environments. It was also evidenced by Matoba and Ogrydziak (1998) that spatial conformation of a SP is another key factor which influences the secretory rate. As demonstrated, a bend introduced by a proline residue directly after the cutting site by a dipeptidase enabled co-translational translocation of AEP protein, while upon elimination of the proline-driven bend, the protein was not translocated. This in turn could be partially alleviated by increasing hydrophobicity of the SP. Ultimately, it was evidenced that the targeting pathway preference and secretory potential of a given SP can be engineered by mutations that have little or no effect on signal peptide hydrophobicity but rather on spatial conformation, and that bended polypeptides with larger radius of gyration interact more readily with SRP, while for more linear SPs, the affinity to SRP can be engineered by increasing hydrophobicity value. Still the authors stated that there have to be some further, unidentified factors driving the secretory potential of the SPs, like amphiphilicity, hydrophobic moment, molecular hydrophobicity potential, or slower rate of synthesis, allowing for longer interaction with SRP (Matoba and Ogrydziak 1998). The latter statement greatly corresponds with the data obtained in this study, as we were able to identify superior and inferior SPs, either if fused with SoAMY or TlGAMY, suggesting a kind of universal observation on these SP performances in *Y. lipolytica* cells; however, we could not find any positive correlation between experimental data on secretory efficiency and in silico calculated *D* score or GRAVY values.

With respect to the predicted secondary structure, the SSP9, characterized by the lowest hydrophobicity value, was the least complying with the general SP structure out of the SPs under study. As shown by SOPMA analysis, SP9 was interrupted by extended strands, random coils, but most importantly—by beta turns, making it a special case of SP having beta turn prior to the C-terminal domain (together with SP3 upon cloning with TlGAMY). Moreover, SP9, unlike the other SPs, contains glycine (G) residues within the core region, suggesting that the structure is rather flexible. While SP9 operated relatively poorly with SoAMY, it functioned better with its native protein. The major difference in the N-terminal region structure between the two proteins is the presence of a proline (P) residue directly after the cutting site, which applies to all the variants with TlGAMY. This in turn results in uniform formation of a random coil structure at the end of SPs preceding the mature TlGAMY protein, which were all scored higher values upon SignalP evaluation (higher *D* scores). The importance of the proline presence immediately after the cutting site for translocation of the polypeptide was demonstrated by Matoba and Ogrydziak (1998), as discussed above. SPs that performed best in secretion of TlGAMY, SP3 and SP4, were terminated with random coil and beta turn structures. Helical structures of SP5 and SP6 cloned with TlGAMY, demonstrating the weakest secretory capacity, were terminated with random coils. However, the same structure was predicted for SP8, SP7, SP1, and also SP10, which, in the three former cases, operated relatively well with this protein, altogether suggesting no straightforward rule for the observed phenomena. On the other hand, when the SPs analyzed in this study were cloned with SoAMY protein, the C-region of the SPs remained in a helical form in most variants, ended with a relatively large and rigid glutamine (Q) residue followed by lysine (K). Beta turn structure in the C-terminus of the SPs followed by SoAMY was identified for SP2 and SP4, the two SPs driving high secretion of the protein. The other analyzed variants of SP-SoAMY constructions, including SP1, SP3, SP6, SP7, and SP10, were all terminated by alpha-helical structure directly prior to the cutting site and drove the secretion with variable strength. The SP5 and SP9, terminated with random coil, functioned relatively poorly with SoAMY, when compared to the other SPs under study.

Finally, the amino acid sequence of the most potent SPs (SP1, SP2, SP3, SP4, SP7, SP8), driving the secretion of the two proteins under study with the highest efficiency, was aligned and a consensus sequence was inferred from this alignment (Table 1.B). Based on this analysis, the following SP consensus sequence was determined: MKFSAALLTAALA(S:V)AAAAA (a sequence of overrepresented amino acid residues). The computed hydrophobicity values for this synthetic SP ranged between 1.468 and 2.158, depending on the S14 or V14 variant, and the extent of the analyzed stretch; however, secondary structure was 100% alpha-helical, which may suggest preference of *Y. lipolytica* towards such SPs. Calculated *D* score values (~ 0.7) were comparable irrespective of the following polypeptide, especially for V14 variant. The consensus sequence determined for the 6 best SPs well corresponded with the consensus sequence determined for the 38 proteins (deduced from the in silico proteome analyses; MKFSTILL(A:L)AA(A:L)(A:V)(A:L)(A:L)AA-P; Table 1.A.) considering the properties of the amino acid residues building the SP. Importantly, the consensus sequence of SP derived from the six best SPs was characterized by much lower degeneration at individual sites of the consensus SP.

In conclusion, based on the adopted strategy, we were able to (i) identify novel, previously undescribed in this context SPs, selected from amongst the complete secretome of *Y. lipolytica*, (ii) characterize their secretory capacity with respect to two model proteins (heterologous amylolytic enzymes), (iii) compare the novel SPs with those previously described and frequently exploited in secretory expression of heterologous proteins in *Y. lipolytica*, (iv) indicate the most potent SPs to be adopted as building blocks in the molecular toolbox for engineering *Y. lipolytica*, and (v) suggest a consensus sequence for potentially robust synthetic SP to be used in secretory overexpression in *Y. lipolytica*.

## Electronic supplementary material

ESM 1 (PDF 1435 kb).
