# Supplementary material for: Robust signal peptides for protein secretion in Yarrowia lipolytica: identification and characterization of novel secretory tags
Source: Appl Microbiol Biotechnol. 2018 Apr 27;102(12):5221–33. doi: 10.1007/s00253-018-8966-9 (PMC5959983; doi:10.1007/s00253-018-8966-9)
Supplement: Supplementary file 1 — (PDF 1435 kb). [file 253_2018_8966_MOESM1_ESM.pdf]

Online Resources

Applied Microbiology and Biotechnology

Robust signal peptides for protein secretion in *Yarrowia lipolytica*: identification and characterization of novel secretory tags.

Ewelina Celińska*[1], Monika Borkowska[1], Wojciech Białas[1], Paulina Korpys[1], Jean-Marc Nicaud[2]

[1]Department of Biotechnology and Food Microbiology, Poznan University of Life Sciences, ul. Wojska Polskiego 48, 60-627 Poznań, Poland

[2]Micalis Institute, INRA-AgroParisTech, UMR1319, Team BIMLip: Integrative Metabolism of Microbial Lipids, Domaine de Vilvert, 78352 Jouy-en-Josas, France

*Corresponding author: Ewelina Celińska, phone: +48618466007, e-mail: ewelina.celinska@up.poznan.pl, ORCID 0000-0001-8372-8459

**Online Resource ESM_1**. *E. coli* strains and bacterial plasmids used in this study				
***E. coli* JM109**	characteristics: *F'(traD36,proAB+,lacIq,Δ(lacZ)M15), endA1,recA1,hsdR17(rk-mk+), mcrA,supE44,λ, gyrA96,relA1, Δ(lacproAB),thi-1*			
All the following strains are descendants of JM109 and bear the corresponding GGVs (TOPO backbone + insert)				
***E. coli* strain No**	**Type and signature of the GG element**	**Backbone Vector**	**Resistance**	**Insert type**
GGE 0065	GGV0065	TOPO	KanR	SSP 1
GGE 0066	GGV0066	TOPO	KanR	SSP 2
GGE 0063	GGV0063	TOPO	KanR	SSP 3
GGE 0067	GGV0067	TOPO	KanR	SSP 4
GGE 0068	GGV0068	TOPO	KanR	SSP 5
GGE 0069	GGV0069	TOPO	KanR	SSP 6
GGE 0070	GGV0070	TOPO	KanR	SSP 7
GGE 0071	GGV0071	TOPO	KanR	SSP 8
GGE 0072	GGV0072	TOPO	KanR	SSP 9
GGE 0062	GGV0062	TOPO	KanR	SSP 10
GGE 0073	GGV0073	TOPO	KanR	SoAMY_no ATG_BsaI_del
GGE 0064	GGV0064	TOPO	KanR	TlGAMY_no ATG
GGE 0003	GGV0003	TOPO	KanR	P1 TEF
GGE 0016	GGV0016	TOPO	KanR	T3 Lip2
GGE 0013	GGV0013	TOPO	KanR	M *URA3ex*
GGE 0037	GGV0037	TOPO	KanR	InsUP_zeta NotI
GGE 0038	GGV0038	TOPO	KanR	InsDOWN_zeta NotI
GGE 0029		pSB1A3-RFP	AmpR	pSB1A3-RFP (iGEM collection)
*All the following strains are descendants of JM109 and bear the following GGVAs (pSB1A3 backbone + GGA with the following constant elements: InsUP-zeta_NotI_ M_URA3ex_ pTEF_ *variable insert* _T3 tLip2_InsDOWN_zeta_NotI)*				
***E. coli* strain No**	**Type and signature of GG element**	**Backbone Vector**	**Resistance**	***variable insert* type**
GGE 0076	GGVA0076	pSB1A3	AmpR	SSP1 TlGAMY
GGE 0078	GGVA0078	pSB1A3	AmpR	SSP2 TlGAMY
GGE 0079	GGVA0079	pSB1A3	AmpR	SSP3 TlGAMY
GGE 0080	GGVA0080	pSB1A3	AmpR	SSP4 TlGAMY
GGE 0081	GGVA0081	pSB1A3	AmpR	SSP5 TlGAMY
GGE 0082	GGVA0082	pSB1A3	AmpR	SSP6 TlGAMY
GGE 0083	GGVA0083	pSB1A3	AmpR	SSP7 TlGAMY
GGE 0084	GGVA0084	pSB1A3	AmpR	SSP8 TlGAMY
GGE 0085	GGVA0085	pSB1A3	AmpR	SSP9 TlGAMY
GGE 0086	GGVA0086	pSB1A3	AmpR	SSP10 TlGAMY
GGE 0087	GGVA0087	pSB1A3	AmpR	SSP1 SoAMY
GGE 0088	GGVA0088	pSB1A3	AmpR	SSP2 SoAMY
GGE 0089	GGVA0089	pSB1A3	AmpR	SSP3 SoAMY
GGE 0090	GGVA0090	pSB1A3	AmpR	SSP4 SoAMY
GGE 0091	GGVA0091	pSB1A3	AmpR	SSP5 SoAMY
GGE 0092	GGVA0092	pSB1A3	AmpR	SSP6 SoAMY
GGE 0093	GGVA0093	pSB1A3	AmpR	SSP7 SoAMY
GGE 0094	GGVA0094	pSB1A3	AmpR	SSP8 SoAMY
GGE 0095	GGVA0095	pSB1A3	AmpR	SSP9 SoAMY
GGE 0096	GGVA0096	pSB1A3	AmpR	SSP10 SoAMY

Online Resource ESM_2. *Y. lipolytica* strains and expression cassettes used in this study

***Y. lipolytica* Po1g**	*Genotype: MatA, leu2-270, ura3-302::URA3, xpr2-332, axp-2*<br>*Po1g is a derivative of the wild-type strain W29 (ATCC 20460; CLIB89); part of YLEX Expression Kit (Yeastern Biotech Co. Ltd., Taiwan)*

The following strains were transformed with pYLSC vector bearing respective gene of interest devoid of its native SP
pBR322 backbone, hybrid promoter (hp4d), XPR2 pre-leader, LEU2—selection marker;
SoAMY and TlGAMY genes were cloned in SfiI/KpnI sites
http://www.yeastern.com/Products.php?pid=273 (Yeastern Biotech Co. Ltd., Taiwan)

***Y. lipolytica* 1.1.2**	*Po1g derivative bearing pYLSC-SoAMY construct*<br>*Used as a positive control in microSIT assay*
***Y. lipolytica* 2.1.2**	*Po1g derivative bearing pYLSC-TlGAMY construct*<br>*Used as a positive control in microSNT assay*
***Y. lipolytica* JMY2101**	*Genotype: MATa, ura3-302, xpr2-322+pXPR2-SUC2*<br>*JMY2101 is a derivative of the wild-type strain W29 (ATCC 20460; CLIB89)*

The following strains were transformed with corresponding GGA (GGA released from pSB1A3 backbone) and contain the following constant elements integrated with the genome:
*InsUP-zeta_NotI_ M_Ura3ex_ pTEF_ *variable insert* _T3 tLip2_InsDOWN_zeta_NotI*

***Y. lipolytica* strain No**	**Type and signature of the GG element**	***variable insert* type**
GGY01	GGA0076	SSP1 TlGAMY
GGY02		
GGY03		
GGY04		
GGY05		
GGY10	GGA0078	SSP2 TlGAMY
GGY11		
GGY12		
GGY13		
GGY14		
GGY24	GGA0079	SSP3 TlGAMY
GGY27		
GGY29		
GGY30		
GGY35		
GGY37	GGA0080	SSP4 TlGAMY
GGY39		
GGY40		
GGY41		
GGY42		
GGY45	GGA0081	SSP5 TlGAMY
GGY46		
GGY48		
GGY49		
GGY50		
GGY60	GGA0082	SSP6 TlGAMY
GGY61		
GGY68		
GGY65		
GGY67		
GGY70	GGA0083	SSP7 TlGAMY
GGY71		
GGY72		
GGY73		
GGY77		
GGY97	GGA0084	SSP8 TlGAMY
GGY98		
GGY101		
GGY102		
GGY103		
GGY125	GGA0085	SSP9 TlGAMY
GGY126		
GGY128		
GGY130		

GGY131		
GGY155	GGA0086	SSP10 TlGAMY
GGY156		
GGY157		
GGY159		
GGY160		
GGY172	GGA0087	SSP1 SoAMY
GGY173		
GGY175		
GGY183		
GGY195		
GGY207	GGA0088	SSP2 SoAMY
GGY208		
GGY212		
GGY220		
GGY226		
GGY255	GGA0089	SSP3 SoAMY
GGY257		
GGY262		
GGY264		
GGY270		
GGY304	GGA0090	SSP4 SoAMY
GGY306		
GGY310		
GGY314		
GGY316		
GGY331	GGA0091	SSP5 SoAMY
GGY332		
GGY337		
GGY338		
GGY339		
GGY345	GGA0092	SSP6 SoAMY
GGY346		
GGY347		
GGY342		
GGY343		
GGY352	GGA0093	SSP7 SoAMY
GGY353		
GGY356		
GGY359		
GGY364		
GGY370	GGA0094	SSP8 SoAMY
GGY373		
GGY380		
GGY383		
GGY389		
GGY394	GGA0095	SSP9 SoAMY
GGY395		
GGY397		
GGY400		
GGY403		
GGY412	GGA0096	SSP10 SoAMY
GGY413		
GGY414		
GGY417		
GGY418		

Online Resource ESM_3. Synthetic Golden Gate Primers (GGPs) and Golden Gate Fragments (GGFs) used in this study

**GGPs**		Comments
**Amplification of constant elements of GGAs**		
GGP_InsertUP_zeta_A_F_Not	GGTCTCTGCCTGCGGCCGCtgtcgggaaccgc	Amplification of GGFs
GGP_InsertUP_zeta_B_R	GGTCTCTACCTtctagcaaagtgctttgtgc	
GGP_M_Ura3ex_B_F	GGTCTCTAGGTataacttcgtatagcatacattatacgaag	
GGP_M_Ura3ex_C_R	GGTCTCTAGCTtcgcttcggataactcc	
GGP_P1_TEF_C_F	GGTCTCTAGCTgggttggcggcg	
GGP_P1_TEF_D_R	GGTCTCTCATTcttcgggtgtgagttgac	
GGP_T3_LIP2_E_F	GGTCTCTGTACgtgtctgtggtatctaagctatttatc	
GGP_T3_LIP2_L_R	GGTCTCTACTCcgatttgtcttagaggaacgc	
GGP_InsertDOWN_zeta_L_F	GGTCTCTGAGTcatgtgtaacactcgctctg	
GGP_InsertDOWN_zeta_M_R_Not	GGTCTCTCGCAGCGGCCGCactgaagggctttgtgag	
**Amplification of a gene in G1 position**		
GGP_G_SoAMY_noSP_x_F	GGGGGTCTCtTGCCCAGAAGGACCCCCAC	
GGP_G_SoAMY_noSP_K_R	GGGGGTCTCtGTACCTAGTGGTGGTGGTGG	Amplification of variable insert R
GGP_G_TlGAMY_noSP_x_F	GGGGGTCTCtTGCCAGACCAGAAACAACCGTTC	
GGP_G_TlGAMY_noSP_K_R	GGGGGTCTCtGTACTCAATGATGATGATGATGATGTCTC	Amplification of variable insert R
**BsaI deletion**		
SoAMY BsaI del noSP 1 F	CAGAAGGACCCCCACTTCCTAGATGGACGAAAC	Deletion of BsaI site from SoAMY gene sequence
SoAMY BsaI del 1 R	CCAGTTGGTCAGCTCAGTTCCCGtGtgCACGTTTCG	
SoAMY BsaI del 2 F	CATGGTTGAGTTTCGAAACGTGcaCaCGGGAAC	
SoAMY BsaI del 2 R	CTAGTGGTGGTGGTGGTGGTGCAGCTTAGCG	
**Amplification of variable insert**		
GGP_SP_B03564g_D_F	ATGAAGTTCACATTTGCTGCCGTTACCGCCGCGCTGGCCTCGTCCGCCATTGCC	Amplification of variable insert F
GGP_SP_D20680g_D_F	ATGAAGTTCTCCACCGCCCTTCTGGCTCTGGCCGCCGTCGCCACTGCC	
GGP_SP_E22374g_D_F	ATGAAATCTCTATTGCTGTCGCTGCTGGCGGTCCCGGCCACTGCC	
GGP_SP_D06039g_D_F	ATGAAGTTCTCAGCGGTCTCAATCGCTGCTGCCCTGGCCTCGCTGGTGGCTGCC	
GGP_SP_D06149g_D_F	ATGAAGCTCTCCATCGTTCTCGTGGCTCTGGCAGCCGTCTCCTCTGCC	
GGP_SP_Lip2NATIVE_D_F	ATGAAGCTTTCCACCATCCTTTTCACAGCCTGCGCTACCCTGGCTGCTGCC	
GGP_SP_Lip2pre 3xXA_D_F	ATGAAGCTGTCTACCATTCTGTTTACCGCTTGTGCTACTCTGGCTCTCGCTCTTGCC	
GGP_SP_SoAMY NATIVE_D_F	ATGAAGGTGCTCGCCCTGCTGGTTACTGTCTGCTTTTCCGTTGCCTCTGCC	
GGP_SP_TlGAMY NATIVE_D_F	ATGTTATTCCAACCGACTTTGTGCGCGGCCCTTGGACTCGCCGCCTTGATCGTCCAAGGCGGAGATGCC	
GGP_SP_XPR2pre_D_F	ATGAAGCTCGCTACCGCCTTTACTATTCTCACGGCCGTTCTTGCC	
**GGFs**		

SoAMY nt no SP		CAGAAGGACCCCCACTTCCTAGATGGACGAAACACTATCGTTCACCTCTTCGAGTGGAAGTGGGCCGACATTGCTTCCGAGTGTGAGAACTTCCTGTCGGTGAAGAACTTTGCC<br>GGTGTTCAGGTGTCTCCCCCTGCTGAGTCCGTGGTCGTTGAGGGCCGACCCTGGTGGGAGAAGTACCAGCCTGTCTCCTACACCCTCAACAACCGAGGCGGAGACGAGGCCGC<br>TTTCTCCGATATGGTTTCGCGATGTAACAACGTGGGAATCCGAATTTACGTGGACCTGGTCGCCAACCACATGGCTACTTCGAACGGCCAGGGATCTGCCGGTAACACCTGCGAC<br>CCCTCTTCCAAGTCTTACCCTGCTGTCTCGTACACTTCTGAGAACTTCCATACCTCCTGTGACATCGATTACACTGACTCGTCTTCCATTCGAAACTGCGAGCTGACCGGCCTCAAG<br>GACCTGGATCAGTCCCAGGACTACGTGCGAGGAAAGATCGAGGAGTACATGAACCACCTCATTTCTCTGGGAGTGGCCGGTTTTCGAGTGGACGCCGCTAAGCATATGTGGCC<br>CGCCGACCTCCAGGCTATCTTCGGCTCCCTCAACGACCTGTCTACCGACCACGGATTTGCTTCGGGTGCCCGAGCTTTCATCTTTCAGGAAGTGATTGACACTTCTACCGATCCTG<br>TCAAGAACACTGAGTACACCGGTTTCGGCAAGGTGTGCGAGTTCCTCTTTGGCAACGACCTGGGACCCGCCTTTCGAGGAGAGAACCCTCTCCATTACCTGAAGAACTGGGGTA<br>CTGAGTGGGGCCTGCTCGACGGTGGCGATACCGTTTCGTTCGTGGACAACCACGATAACGAGCGAGACTCTCAGATTTTCCTGCATTACACCAACGATAAGCCCTACAAGGCTG<br>CCATGGCCTTCATGCTCGCTCACTCCTACGACACCACTACCCGAGTCCTGTCGTCTTACAAGTTTGACTCCTCGGATCAGGGTCCCCCTTCTAACGGCGACGATATTCTGTCTCCCG<br>AGTTCGGCTCCGACGGAGCCTGTACCAACGGCTGGGTTTGCCAGCATCGATGGTCCCCTGTCTTCAACATGGTTGAGTTTCGAAACGTGGTCTCGGGAACTGAGCTGACCAACT<br>GGTGGGACAACGGTTCGCAGCAGATTGCCTTCTCTCGAGGTGACAAGGGCTTTTACGCCGCTACTGTCAACGAGGATATCGCTACCTCTATTACTACCGGACTGCCCGACGGTTC<br>CTACTGTGATGTCATGTCTGGCTCTCTGGTGAACGGCGCCTGCACCGGAAAGACTCTGACCGTGTCTGGCGGTCAGGTCTACGTTGAGCTGGGTGGTGCTGAGCTGGAGGCTGC<br>TGTCGCTATTCACGTCAACGCTAAGCTGCACCACCACCACCACCACTAG	codon optimized sequence encoding SoAMY truncated at 5' end to eliminate its native SP
TlGAMY nt no SP		AGACCAGAAACAACCGTTCCTCACGCAACCGGTTCTTTGGATGATTTCTTGGCTGCACAATCACCAATCGCTTTCCAAGGTATTTTGAACAACATTGGTCCATCTGGTGCATATTC<br>AGAAGGTGTTAATCCAGGTGTTGTTATTGCTTCACCATCTAAGCAAGATCCAGATTACTTCTACACTTGGGTTAGAGATGCTGCATTGACAGTTCAATATTTGGTTGAAGAATTGG<br>TTGCTGGTAATGCTTCTTTGCAATTCTTGATCCAAGATTACATCTCTTCACAAGCTAGATTGCAAACTGTTGAAAATCCATCAGGTTCTTTGTCTTCAGGTGGTTTAGGTGAACCAA<br>AGTTCCATGTTGATGAAACTGCTTTTACTGATTCTTGGGGTAGACCACAAAGAGATGGTCCACCATTGAGAGCTATTGCAATGATCTCTTTCGCTAACTATTTGATCGATAACGGT<br>CATCAATCAACAGTTGAAGATATCATCTGGCCAATCGGTAGAAACGATTTGTCATACGTTTCTCAACATTGGAACGAAACTACATTCGATATCTGGGAAGAAGTTCATTCTTCATC<br>TTTCTTTACTACAGCAGTTCAATATAGAGCTTTAGTTCAAGGTTCTGCTTTGGCATCAAAGTTGGGTCATACTTGTGATAACTGTGGTTCTCAAGCACCACAAATTTTGTGTTTCTT<br>GCAATCTTACTGGACTGGTTCACATATTTTGGCTAATACTGGTGGTGGTAGATCTGGTAAAGATGTTTCAACTATTTTAGGTGTTATTGGTTCTTTCGATCCAAATGCTGATTGTG<br>ATGATGTTACATTTCAACCATGTTCAGCTAGAGCATTAGCTAACCATAAGCAAGTTGTTGATTCTTTTAGATCTATCTATGCAATTAATGCTGGTATTCCATCAGGTTCTGCAGTTG<br>CTGTTGGTAGATATCCAGAAGATGTTTACCAAGGTGGTCATCCATGGTACTTGACTACAGCTGCAGCTGCAGAACAATTGTACGATGCAATCTATCAATGGAACCATGTTGGTCA<br>TATCGATATCAACGCTGTTAATTTGGATTTCTTTAAATCTATCTATCCATCAGCTGCAGAAGGTACATACACTTCTGATTCATCTACTTTCCAAGATATCATCTCAGCAGTTAGAACA<br>TATGCTGATGGTTTCTTGTCTGTTATTGAAAAGTACACTCCACCAGATAATTTGTTGGCTGAACAATTCCATAGAGAAACAGGTATTCCATTGTCAGCTGCATCTTTAACTTGGTCT<br>TATGCTGCATTGAATACAGCTGCACAAAGAAGAGCTTCTATTGTTCCATCACCATGGAACTCAAACTCTACAGATTTGCCTGATAAGTGTTCAGCAACTTCTGCTACAGGTCCATA<br>CGCAACACCAACTAATACAGCTTGGCCAACTACAACTCAACCACCAGAAAGACCAGCTTGTACTCCACCATCTGAAGTTACTTTGACTTTTAATGCATTGGTTGATACAGCTTTCG<br>GTCAAAACATATATTTGGTTGGTTCTATTCCAGAATTAGGTTCATGGGACCCAGCAAATGCTTTGTTAATGTCAGCAAAATCTTGGACTTCAGGTAATCCAGTTTGGACATTGTCA<br>ATTTCTTTACCAGCTGGTACTTCTTTCGAATACAAGTTTATTAGAAAGGATGATGGTTCATCTGATGTTGTTTGGGAATCTGATCCAAATAGATCATACAATGTTCCAAAAGATTGC<br>GGTGCTAACACAGCTACAGTAAACTCTTGGTGGAGACATCATCATCATCATCATTGA	codon optimized sequence encoding TlGAMY truncated at 5' end to eliminate its native SP
GGF_SP_B03564g_D_F	SSP_1	GGGGGTCTCtAATGAAGTTCACATTTGCTGCCGTTACCGCCGCGCTGGCCTCGTCCGCCATTGCCaGAGACCCCC	F elements to be aligned with their complementary strand R – not for amplification
GGF_SP_D20680g_D_F	SSP_2	GGGGGTCTCtAATGAAGTTCTCCACCGCCCTTCTGGCTCTGGCCGCCGTCGCCACTGCCaGAGACCCCC	
GGF_SP_E22374g_D_F	SSP_3	GGGGGTCTCtAATGAAATCTCTATTGCTGTCGCTGCTGGCGGTCCCGGCCACTGCCaGAGACCCCC	
GGF_SP_D06039g_D_F	SSP_4	GGGGGTCTCtAATGAAGTTCTCAGCGGTCTCAATCGCTGCTGCCCTGGCCTCGCTGGTGGCTGCCaGAGACCCCC	
GGF_SP_D06149g_D_F	SSP_5	GGGGGTCTCtAATGAAGCTCTCCATCGTTCTCGTGGCTCTGGCAGCCGTCTCCTCTGCCaGAGACCCCC	
GGF_SP_Lip2NATIVE_D_F	SSP_6	GGGGGTCTCtAATGAAGCTTTCCACCATCCTTTTCACAGCCTGCGCTACCCTGGCTGCTGCCaGAGACCCCC	
GGF_SP_Lip2pre 3xXA_D_F	SSP_7	GGGGGTCTCtAATGAAGCTGTCTACCATTCTGTTTACCGCTTGTGCTACTCTGGCTCTCGCTCTTGCCaGAGACCCCC	
GGF_SP_SoAMY NATIVE_D_F	SSP_8	GGGGGTCTCtAATGAAGGTGCTCGCCCTGCTGGTTACTGTCTGCTTTTCCGTTGCCTCTGCCaGAGACCCCC	
GGF_SP_TlGAMY NATIVE_D_F	SSP_9	GGGGGTCTCtAATGTTATTCCAACCGACTTTGTGCGCGGCCCTTGGACTCGCCGCCTTGATCGTCCAAGGCGGAGATGCCaGAGACCCCC	
GGF_SP_XPR2pre_D_F	SSP_10	GGGGGTCTCtAATGAAGCTCGCTACCGCCTTTACTATTCTCACGGCCGTTCTTGCCaGAGACCCCC	
GGF_SP_B03564g_x_R	SSP_11	GGGGGTCTCtGGCAATGGCGGACGAGGCCAGCGCGGCGGTAACGGCAGCAAATGTGAACTTCATTaGAGACCCCC	R elements to be aligned with their complementary
GGF_SP_D20680g_x_R	SSP_12	GGGGGTCTCtGGCAGTGGCGACGGCGGCCAGAGCCAGAAGGGCGGTGGAGAACTTCATTaGAGACCCCC	
GGF_SP_E22374g_x_R	SSP_13	GGGGGTCTCtGGCAGTGGCCGGGACCGCCAGCAGCGACAGCAATAGAGATTTCATTaGAGACCCCC	
GGF_SP_D06039g_x_R	SSP_14	GGGGGTCTCtGGCAGCCACCAGCGAGGCCAGGGCAGCAGCGATTGAGACCGCTGAGAACTTCATTaGAGACCCCC	

GGF_SP_D06149g_x_R	SSP_15	GGGGGTCTCtGGCAGAGGAGACGGCTGCCAGAGCCACGAGAACGATGGAGAGCTTCATTaGAGACCCCC	strand F – not for amplification
GGF_SP_Lip2NATIVE_x_R	SSP_16	GGGGGTCTCtGGCAGCAGCCAGGGTAGCGCAGGCTGTGAAAAGGATGGTGGAAAGCTTCATTaGAGACCCCC	
GGF_SP_Lip2pre 3xXA_x_R	SSP_17	GGGGGTCTCtGGCAAGAGCGAGAGCCAGAGTAGCACAAGCGGTAAACAGAATGGTAGACAGCTTCATTaGAGACCCCC	
GGF_SP_SoAMY NATIVE_x_R	SSP_18	GGGGGTCTCtGGCAGAGGCAACGGAAAAGCAGACAGTAACCAGCAGGGCGAGCACCTTCATTaGAGACCCCC	
GGF_SP_TlGAMY NATIVE_x_R	SSP_19	GGGGGTCTCtGGCATCTCCGCCTTGGACGATCAAGGCGGCGAGTCCAAGGGCCGCGCACAAAGTCGGTTGGAATAACATTaGAGACCCCC	
GGF_SP_XPR2pre_x_R	SSP_20	GGGGGTCTCtGGCAAGAACGGCCGTGAGAATAGTAAAGGCGGTAGCGAGCTTCATTaGAGACCCCC	

Online Resource ESM_4. **(A)** A list of the 54 polypeptides identified through *Yarrowia lipolytica* CLIB122 genomic sequence-mining bearing the motif: MK x{7-15} (x,A/P){2-10} x{10-120} KR. Blue – pre-leader, purple - XA/XP dipeptide motif, black – pro-leader, red – KR dipeptide; the names of the 16 polypeptides eliminated from further analysis (alignment and consensus sequence determination) are crossed out. **(B)** The output data from SignalP and **(C)** Multiple Sequence Alignment (ClustalW) run with the remaining 38 polypeptides.

(A)

>YALI0A00154g
MKLSNIFALATVALAAPQPLITPPPTAVEKRDDQAVLGADVWIQTMPNGHEKR
>YALI0A08360g
MKPSVIIALAVACMAAPMSPQGAYKDALDIINGQASKFNDAGLKKR
>YALI0A09262g
MKPSVILSLAVACLAAPMAPQGAYKDALDIINGQSSKFSDSSLRKR
>YALI0A10208g
MKLLSILLWISIGLCAAIPQLENDYAKLLSLMEHLEKR
>YALI0A11198g
MKFSAIAIAAAASIVVADPIFLGKPAAGGVDPNTFWFSQSQTPTPAPPPPAPTTQAPPPPPAPTSSQAAQETCIAPASTNEKR
~~>YALI0A17534g~~
MKIPGTVPLVESTEIILSPTPSSDPDDPLNWSPRRKLLSMFCMVLYCVAFCVPSASIFSVFIPISQNTGLPLATLNQGTGYMFLLFGLGCIIVHPLSVKYGKR
>YALI0A20350g
MKLSTILFTACATLAAALPSPITPSEAAVLQKR
~~>YALI0A20790g~~
MKFSLKKSTTQTPDSPPPEEEETLAEEPTTDPAIRTFSLGDVVLTDVVSCKDELKEVHMGYLNDQCYIVIDNFAPTLLSPKSQYVHIMAGRIKFTISEEMTRMADRAFLVLTETLEPPKKR
~~>YALI0B03564g~~
MKFTFAAVTAALASSAMALGGLGVDLGVKR
>YALI0B05104g
MKLSTIAISAILGSVALAAPAQEELRSTAMLIQQSEEFQRLNYDEGLLSLPDRDELRLVETAPGVRTWMTEAEKLVLKR
~~>YALI0B07293g~~
MKKQNLNALYPHPYPYVVDVVPPVIHNPISVLYFLCRWIFAGNTSPEYTAEYVDGVVRAVGDAREGLWKMGFFGKGILSRSEPSWDIRTKRRLGLHVEEGVYAPEEVTQFRREQRQHFKAERAKAEEEELKR
>YALI0B08778g
MKVAHIIQLAAMVATALAAVLAIDYGQEYTKAALLSPGINFEIVLTQDSKRKQPSAIGFKGKADSKFGLERVYGSPAVLMEPRFPSDVVLYHKR
~~>YALI0B09713g~~
MKRKMEEDIPTPTEPPSAKQPRQSMARSVSACVRCRKRKSKCDQKFPSCSRCLAAGVVCMGLDAATKR
>YALI0B15510g
MKLSTLVLAALTAVAVADDYKSISCDVDNHCPEDKPCCSQYGECGTGAYCLGGCDPKSSFELKSCMPMPVCKKR
>YALI0B16500g
MKFNPVISAVAMAALAQALLIPQNVDALEAFNTLLTNDVEANSPNHHKAAKFEKIVIANEDESEIAPLVMNDKEQVPDSYIVVFKEDVDASTAASHELWVEQVHTEQVASLAKR
>YALI0B22880g
MKFTLLLAGLSLVAAAPADADASAYSNVLNIINKHQGHHAKR
~~>YALI0C14564g~~
MKILTIDVFGTLFVPRPSVPAQYLRIVQQHEKCSATVAQVQAGFHKAFKR
>YALI0C15851g
MKSAIIGYMAVAVAAASCQFTVDSKNYDLSAISGPKSVEYTIETPPSKR
>YALI0D00759g
MKTSHITVALLAAVATAATIRVDSGNAVTLDIVKR
>YALI0D02024g
MKLTTTTLLALSSLASAFRPFSGHDEIAKAARENVPSLGEPQAAILPKSPFKKAASSPLQNKASSGGTSVATSSVDCKRFVPIEQDGGDVSYYVNTQLGSSQAEYRMVLDTGSYYMWVYSENCTSTACMKR
>YALI0D02981g
MKLATSLTLFTAVLAAPLAAPAPDVAPAVAEGSASAAYSAILSVVSKQSSKFSALQKR
>YALI0D04851g
MKFSTLSVAAALAASASAALDPVEVKGNAFFVGDKR
>YALI0D06039g
MKFSAVSIAAALASLVAAADVPSIDIVGNKFFYSNNGSQFYIRGVAYQQDSANETAKETFSDPLSDGDACKR
>YALI0D06149g
MKLSIVLVALAAVSSAQPIVGVYDKRDSAETVQYIHFADDAHVETRDLTKR
>YALI0D08206g
MKFSATILTALAASAVANYAPIKVECPKDVNISRPADGLSHQEADYINERHKKTSKALREYLGGLDIKARGNNTFDTDSFLNETSPKLAIAAAGGGFRAMLVGAGVIAALDKR
>YALI0D20680g

MKFSTALLALAAVATAQPIVGHHAHHQHKR

~~>YALI0D22616g~~

MKHLKSVAIIGGGPSAAPCIKALLAENYFGKITAYEQQPAPGAGVWNYNGLTRPTPNLPCTRDMPAEPIEHKKDGSIIYYNPMYRDLNTNLPHMLMAYKDFPFPEGVDLFPKR

~~>YALI0D25102g~~

MKDLPPTIVTQLQSYPPGAQIQRLVHIGDSHKPLRQQCLEKALAICRQSTRQVEAYERILKR

>YALI0D26554g

MKFSVLLLAGAVLALPTATVVNTEAIDYSTITDLPGSEPTDVSDLEKRADPFKR

>YALI0E00110g

MKIDIALVVLLATAVSAAPPTFSKIQPSPDAIASAAATAKSNHWKDEGYETVKGQVFDKYYQIWLENTNYDKAFGQADLKALTDEGILLTNYWSLTHPSQPNYIAAVSGDYFGDMTDSFKR

~~>YALI0E04785g~~

MKHPYHIVSLSPDGKTMYAAAARQVDRICLETGECKSTVVEESATRTTTKTGDKPAAAKRTKYLPTSVSHVRSLQLSKGGKYLLATTDETKSLVLLDPTTLEVIKKVQYPKR

>YALI0E05423g

MKLLNIFTIATVASAMSLRRQEPISHVEPADTIFHIDPDLLQPHSSFSLSVKVPIPEGGAHYNRLQQRWYVHADPNARRDAESFFKKR

>YALI0E05819g

MKFSIVLPLAGALLAAAGPIQQHPMEGCVPRSVFDLDGCLDTCNDFLVSDSDPEQCLQCLTVYELDNKNTVLSTLEEQGLPMQPEVPVCGVSAPMITPGPAIPVLAKR

~~>YALI0E09757g~~

MKTTRPNLHINLHNSPNPQSCGGAALGRTLSNTSQSSLPSTPMDQTIHTPHGYLSRNNSITSSVATSEDEDGYMSDLNSNPLTSISNQFWPEHDDAFLMHIYYKVVDNPTIAPFVGHTPPSGIIHRVAKDCAKR

>YALI0E16533g

MKFSTIALAAVACLVSAAPAAPVGTGSHGPQSIPEEAIVGGLQGTENEIFVFFNDDESGKQGIAIIDAKKAQEAGFMDPQPDSEVAAGNAKREASPEAWRWFWLPGYGEPNWKRDAMPADMDKEKR

~~>YALI0E21329g~~

MKLYYIGILRPNKPEAVQLASASDLSSYSWFERSTVGQAFTFLAETVIPRTEPGQRQSVEENDYVGHAYLRSDGLGGVIITDQEYPVRVAYTLLNKVLEEYLTKHPKAEWENAKEASSKLAMPELEDYVKR

>YALI0E22374g

MKSLLLSLLAVPATAQLLLDLQGHDTTDPRHQSQNHLTKR

>YALI0E26719g

MKLATAFTILTAVLAAPLAAPAEAFLFLLAGFAAKISASMTNETSDRPLVHFTPNKGWMNDPNGLWYDEKDAKWHLYFQYNPNDTVWGTPLFWGHATSDDLTNWEDQPIAIAPKR

>YALI0E28875g

MKFSAIVSLFAVAVAAQKSIEQIVSEHQERFRLAKR

>YALI0E30415g

MKFSTIALSIMAAVAIAAPIAQEPAAEVPVAPEAPAAPNMDDLPKQLQGLQGVLQNVLFTNDNEQFFIVEDDDGTRSLLIVDTTLIGSEGLAKREANPEAWRWFWLPGYGEPNWKRDAMPADVDKEKR

~~>YALI0E31064g~~

MKFNHSLQFNAVPEWTSEYIAYSQLKKLIYSLERTALGVPNYEDEDEENSTLIREADSNPEDIFKR

>YALI0E32703g

MKFSKTLLLALVAGALAKGEDEICRVEKNSGKEICYPKVFVPTEEWQVVWPDQVIPAGLHVRMDYENGVKEAKINDPNEEVEGVAVAVGEEVPEGEVVIEDLTEENGDEGISANEKVQRAIEKAIKEKR

>YALI0E33539g

MKFSTLALAAMAAIGFAAPATPDADCNEEKEVRDFHAHHQHKR

~~>YALI0E34133g~~

MKAWHWSVTLVVIYLAIPVILYLLTRKDDRKPLSDIRKRKR

~~>YALI0E34852g~~

MKFLVCLLVLALASVVSAHNVLLLPWKR

>YALI0F05390g

MKLTKLVALAGAALASPIQLVPREGSFLGFNYGSEKVHGVNLGGWFVLEPFITPSLFEAFGNNDANVPVDEYHYTAWLGKEEAEKR

>YALI0F05786g

MKFSTVLLTAAVAAAELITLDWAETTQVISIPDATAGEQATSAVLSAAYDAIHDHLAAQTTPAPDAEKAEEALDLTIQAPFFTMGLWIPDLSDFGIGTKNETISEWLHRIWEEKQGVAYPNRKR

>YALI0F11803g

MKTAAILSCLGLVVASAIPDFTTSSQFPTKR

>YALI0F12067g

MKFSNVLLISALSAASVAAPVAEPEPKNVWVPKVPDNVLPGPEKRTLEKR

~~>YALI0F12969g~~

MKIPNVSTPPAVPSLTTKPLTVTKTKPKAKEEPQEPPQDMSLHLQQLMLSTDNVQNNSFSEDFLEQLVPIVKDAIKTKR

>YALI0F19030g

MKFSATLLAAAVSVAVADPILGKGPDPNTFWFSQPSTPGAPAPSNPPAAPAPKPNPGGVDPNTFWFSQSSTPTPAPPAPAPSTQAPAPPASSPAPPASSAAPAPQPEETCIAPAGAQEKR

>YALI0F19646g

MKLSLLLLSTLAVALPIAQQPASEYNQLLDLINNQDLEKR

~~>YALI0F20966g~~

MKTSLQLRRNLPGAAAWVPPDFGKVYFTGLSAVAIGAATKAVVRVSTFTAFSKFMADEDNRTTAPRVVTAGLLTGITESFIVVPFESIKTTMINRVQVAAGKPVAVPPTEIKLPEKPETAAKGPVETAPVTIKDKKR

>YALI0F31251g

MKLPILASLSGLAGLFTTAHAFGAGNIPSDSSIEGKNFRHGDIEDVLAGIVISHRGGKARKFDGLDIKR

(B)

```
SignalP output data
##gff-version 2
##sequence-name  source   feature  start   end     score   N/A ?
## ----------------------------------------------------------
YALI0A00154g     SignalP-4.1      SIGNAL  1       15      0.639   .       .       YES
YALI0A08360g     SignalP-4.1      SIGNAL  1       15      0.513   .       .       YES
YALI0A09262g     SignalP-4.1      SIGNAL  1       15      0.617   .       .       YES
YALI0A10208g     SignalP-4.1      SIGNAL  1       17      0.780   .       .       YES
YALI0A11198g     SignalP-4.1      SIGNAL  1       17      0.586   .       .       YES
YALI0A20350g     SignalP-4.1      SIGNAL  1       17      0.571   .       .       YES
YALI0B05104g     SignalP-4.1      SIGNAL  1       18      0.685   .       .       YES
YALI0B08778g     SignalP-4.1      SIGNAL  1       18      0.618   .       .       YES
YALI0B15510g     SignalP-4.1      SIGNAL  1       17      0.770   .       .       YES
YALI0B16500g     SignalP-4.1      SIGNAL  1       27      0.572   .       .       YES
YALI0B22880g     SignalP-4.1      SIGNAL  1       15      0.767   .       .       YES
YALI0C15851g     SignalP-4.1      SIGNAL  1       15      0.451   .       .       YES
YALI0D00759g     SignalP-4.1      SIGNAL  1       19      0.539   .       .       YES
YALI0D02024g     SignalP-4.1      SIGNAL  1       17      0.702   .       .       YES
YALI0D02981g     SignalP-4.1      SIGNAL  1       15      0.818   .       .       YES
YALI0D04851g     SignalP-4.1      SIGNAL  1       18      0.555   .       .       YES
YALI0D06039g     SignalP-4.1      SIGNAL  1       18      0.820   .       .       YES
YALI0D06149g     SignalP-4.1      SIGNAL  1       16      0.822   .       .       YES
YALI0D08206g     SignalP-4.1      SIGNAL  1       17      0.582   .       .       YES
YALI0D20680g     SignalP-4.1      SIGNAL  1       16      0.600   .       .       YES
YALI0D26554g     SignalP-4.1      SIGNAL  1       23      0.623   .       .       YES
YALI0E00110g     SignalP-4.1      SIGNAL  1       17      0.811   .       .       YES
YALI0E05423g     SignalP-4.1      SIGNAL  1       15      0.490   .       .       YES
YALI0E05819g     SignalP-4.1      SIGNAL  1       17      0.689   .       .       YES
YALI0E16533g     SignalP-4.1      SIGNAL  1       20      0.771   .       .       YES
YALI0E22374g     SignalP-4.1      SIGNAL  1       15      0.755   .       .       YES
YALI0E26719g     SignalP-4.1      SIGNAL  1       24      0.588   .       .       YES
YALI0E28875g     SignalP-4.1      SIGNAL  1       16      0.540   .       .       YES
YALI0E30415g     SignalP-4.1      SIGNAL  1       17      0.736   .       .       YES
YALI0E32703g     SignalP-4.1      SIGNAL  1       17      0.759   .       .       YES
YALI0E33539g     SignalP-4.1      SIGNAL  1       19      0.622   .       .       YES
YALI0F05390g     SignalP-4.1      SIGNAL  1       15      0.570   .       .       YES
YALI0F05786g     SignalP-4.1      SIGNAL  1       23      0.623   .       .       YES
YALI0F11803g     SignalP-4.1      SIGNAL  1       15      0.556   .       .       YES
YALI0F12067g     SignalP-4.1      SIGNAL  1       18      0.697   .       .       YES
YALI0F19030g     SignalP-4.1      SIGNAL  1       17      0.748   .       .       YES
YALI0F19646g     SignalP-4.1      SIGNAL  1       14      0.674   .       .       YES
YALI0F31251g     SignalP-4.1      SIGNAL  1       21      0.641   .       .       YES
```

(C)

Multiple Sequence Alignment ClustalW (MEGA 7.0 Package)

Gene Name	MSA
YALI0A00154g	--MKLSNIFALATVALAAPQP-------
YALI0A08360g	--MKPSVIIALAVACMAAP---------
YALI0A09262g	--MKPSVILSLAVACLAAPMA-------
YALI0A10208g	--MKLLSILLWISIGLCAAIP-------
YALI0A11198g	--MKFSAIAIA-AAASIVVADP------
YALI0A20350g	--MKLSTILFTACATLAAALPSP-----
YALI0B05104g	--MKLSTIAISAILGSVALAAP------
YALI0B08778g	--MKVAHIIQLAAMVATALAAVLA----
YALI0B15510g	--MKLSTLVLA-ALTAVAVA--------
YALI0B16500g	--MKFNPVISAVAMAALAQA--------
YALI0B22880g	--MKFTLLLAGLSLVAAAPADADASA--
YALI0C15851g	--MKSAIIGYMAVAVAAA----------
YALI0D00759g	--MKTSHITVA-LLAAVATA--------
YALI0D02024g	--MKLTT-TTLLALSSLASA--------
YALI0D02981g	--MKLATSLTLFTAVLAAPLAAPAP---
YALI0D04851g	--MKFSTLSVAAALAASASA--------
YALI0D06039g	--MKFSAVSIAAALASLVAAA-------
YALI0D06149g	--MKLSIVLVALAAVSSAQP--------
YALI0D08206g	--MKFSATILT-ALAASAVA--------
YALI0D20680g	--MKFSTALLALAAVATA----------
YALI0D26554g	--MKFSVLLLAGAVLALPTAT-------
YALI0E00110g	MKIDIALVVLLATAVSAAPP--------
YALI0E05423g	--MKLLNIFTIATVASA-----------
YALI0E05819g	--MKFSIVLPLAGALLAAA---------
YALI0E16533g	--MKFSTIALA-AVACLVSAAPAAP---
YALI0E22374g	--MK-SLLLS----LLAVPATA------
YALI0E26719g	--MKLATAFTILTAVLAAPLAAPAEA--
YALI0E28875g	--MKFSAIVSL-FAVAVA----------
YALI0E30415g	--MKFSTIALS-IMAAVAIAAPIA----
YALI0E32703g	--MKFSKTLLL-ALVAGALA--------
YALI0E33539g	--MKFSTLALA-AMAAIGFAAPATPDA-
YALI0F05390g	--MKLTKLVALAG-AALASP--------
YALI0F05786g	--MKFSTVLLTAAVAAA-----------
YALI0F11803g	--MKTAAILSC-LGLVVASAIP------
YALI0F12067g	--MKFSNVLLISALSAASVAAPVAEPEP
YALI0F19030g	--MKFSATLLA-AAVSVAVADP------
YALI0F19646g	--MKLSLLLLS---TLAVALPIA-----
YALI0F31251g	--MKLPILASLSGLAGLFTTAHA-----
